# Transient Osteoporosis of the Hip: A Case Report

**DOI:** 10.7759/cureus.75571

**Published:** 2024-12-11

**Authors:** Asl Abu-Nayla, Ahila Abu-Nayla, Abdulameer Abu Nailah

**Affiliations:** 1 Orthopaedics and Trauma, Gulf Medical University, Ajman, ARE; 2 Intensive Care Unit, Prime Hospital, Dubai, ARE; 3 Rheumatology, Canadian Specialist Hospital, Dubai, ARE

**Keywords:** avascular necrosis (avn), bone marrow edema, diagnosis, magnetic resonance imaging (mri), transient osteoporosis of the hip (toh)

## Abstract

Transient osteoporosis of the hip (TOH), also known as bone marrow edema (BME), is an uncommon condition of unknown etiology. While transient osteoporosis usually affects the hip, it could affect other joints as well. The most common presentation is pain and it has been linked to reduced bone mineral density. This self-limiting disease predominantly affects middle-aged men and pregnant women in their third trimester, presenting unique challenges in diagnosis and management. The exact cause of TOH remains uncertain, with theories suggesting vascular disruption, altered bone remodeling, and neurovascular influences as potential factors.

## Introduction

Transient osteoporosis of the hip (TOH), also referred to as regional migratory osteoporosis, is a rare and poorly understood condition characterized by sudden-onset hip pain and temporary bone demineralization that typically impacts weight-bearing joints. Although different from primary osteoporosis, it shares some similarities in bone density changes; however, it possesses unique clinical characteristics and tends to affect specific demographic groups. This condition predominantly affects middle-aged men and pregnant women in their third trimester, with a male-to-female ratio of 3:1. The abrupt and severe hip pain associated with this condition, exacerbated by weight-bearing activities, can significantly impair mobility and diminish the quality of life for affected individuals [1,2].

Despite extensive research, the underlying mechanisms responsible for the development of TOH remain elusive, with various theories indicating vascular disruption, altered bone remodeling, and neurovascular influences as potential factors. Remarkably, despite its alarming presentation and concerning radiological findings, transient osteoporosis of the hip joint is considered to be self-limiting, typically resolving spontaneously over a few months. Nevertheless, the acute phase of this condition can be distressing for patients, necessitating appropriate management strategies to alleviate symptoms and promote recovery [3,4].

Management approaches for TOH are primarily conservative, aiming to control pain, reduce weight-bearing stress on the affected hip, and facilitate bone healing. The commonly prescribed treatments include rest, analgesics, and physical therapy to improve joint function and alleviate discomfort. In more challenging cases, pharmacological interventions like bisphosphonates, which inhibit bone resorption, may be considered. Moreover, joint-preserving surgeries such as core decompression have been attempted in certain cases, but the efficacy of such procedures remains a topic of ongoing debate [11,12].

## Case presentation

A 58-year-old Jordanian male presented to the rheumatology clinic with a two-month history of low back pain radiating to his left hip joint. The pain was particularly severe in the morning, forcing him to wake up, and was exacerbated by walking and climbing stairs. Previously, the patient had visited the neurosurgery clinic; an X-ray performed there had been normal whereas a lumbosacral MRI had revealed mild lumbar disc prolapse at L5-S1 and spondylolysis. Despite taking pain relievers, the patient's condition had not improved.

On examination, the patient exhibited painful restriction during the external rotation of the hip. Laboratory analysis demonstrated normal values for C-reactive protein (1.06 mg/L), aspartate aminotransferase (14 U/L), calcium levels (9.36 mg/dl), and ferritin levels (116.3 ng/ml). Creatinine levels were also normal (1.20 mg/dl). Lipid profile showed normal non-HDL cholesterol (129 mg/dl), near-optimal levels of LDL cholesterol (128 mg/dl), and high levels of HDL cholesterol (46 mg/dl); triglycerides were normal (65 mg/dl) and cholesterol showed desirable levels (175 mg/dl). Uric acid levels (5.56 mg/dl) and blood urea nitrogen (7.9 mg/dl) were also normal. Complete blood count (CBC) showed elevated mean platelet volume levels (10.1 fL), elevated lymphocytes (3.25 x10^9^), and normal levels of erythrocyte sedimentation count (10 mm/hour). All other lab tests were normal, including renal function tests (electrolytes and bicarbonate); liver function tests (alanine transaminase, alkaline phosphatase, total protein, albumin, and bilirubin); thyroid function tests (thyroid-stimulating hormone and free T4); glucose levels; inflammatory markers (rheumatoid factor and ANA); bone-specific markers (vitamin D and bone-specific alkaline phosphatase); and urine analysis Table 1.

**Table 1:** Laboratory results of the patient Reference values in the table are for a specific gender and age group

Test	Patient value	Normal value
C-reactive protein	1.06 mg/L	0-6 mg/L
Aspartate aminotransferase	14 U/L	12-37 U/L
Calcium levels	9.36 mg/dl	8.8-10.6 mg/dl
Ferritin levels	116.3 ng/ml	30 - 400 ng/mL
Creatinine levels	1.20 mg/dl	0.5-1.2 mg/dl
Non-HDL cholesterol	129 mg/dl	<130 mg/dl
LDL cholesterol	128 mg/dl	<100 mg/dl
HDL cholesterol	46 mg/dl	>40 mg/dl
Triglycerides	65 mg/dl	<150 mg/dl
Total cholesterol	175 mg/dl	<200 mg/dl
Uric acid	5.56 mg/dl	3.4-7.0 mg/dl
Blood urea nitrogen	7.9 mg/dl	6-20 mg/dl
Mean platelet volume	10.1 fL	6.4-9.7 fL
Lymphocytes	3.25 *10^9	1.0-3.0 *10^9
Erythrocyte sedimentation rate	10 mm/hour	0-20 mm/hour

**Figure 1:**
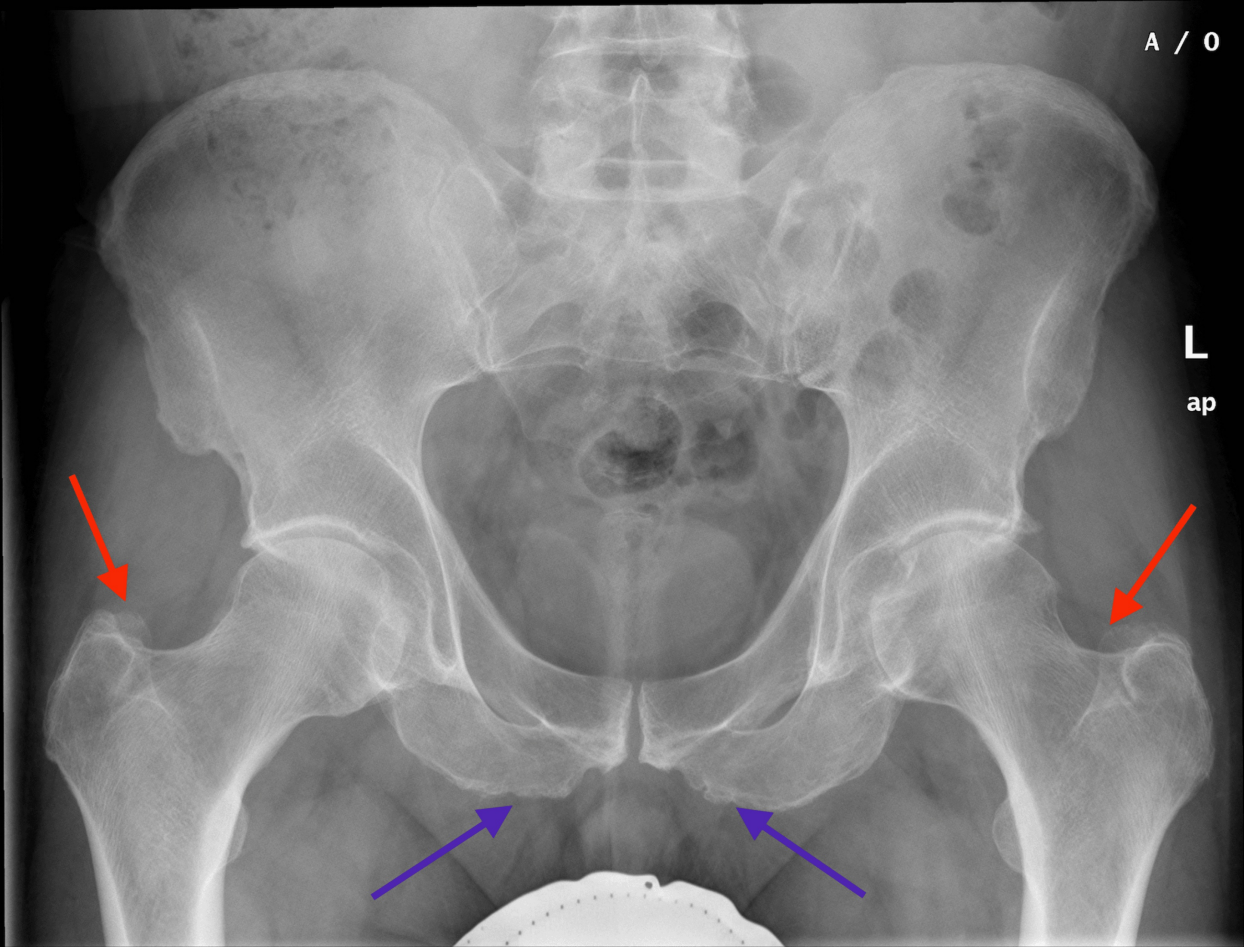
X-ray of the pelvis in the anterior-posterior view Both hip joints showed normal alignment and articular margins. Enthesopathy was seen at bilateral ischial tuberosities (blue arrow) and bilateral supraacetabular prominence (red arrow) was also seen

An X-ray of the pelvis in the anterior-posterior view (Figure 1) and that of the left hip joint in both lateral and anterior-posterior views were taken. Both hip joints showed normal alignment and articular margins. Enthesopathy was seen at bilateral ischial tuberosities and bilateral supra-acetabular prominences were observed. The left hip joint space was slightly decreased inferomedially but was normal superiorly. The right joint space appeared normal, without any fracture noted. Both sacroiliac joints appeared normal and the periarticular soft tissues were unremarkable.

**Figure 2:**
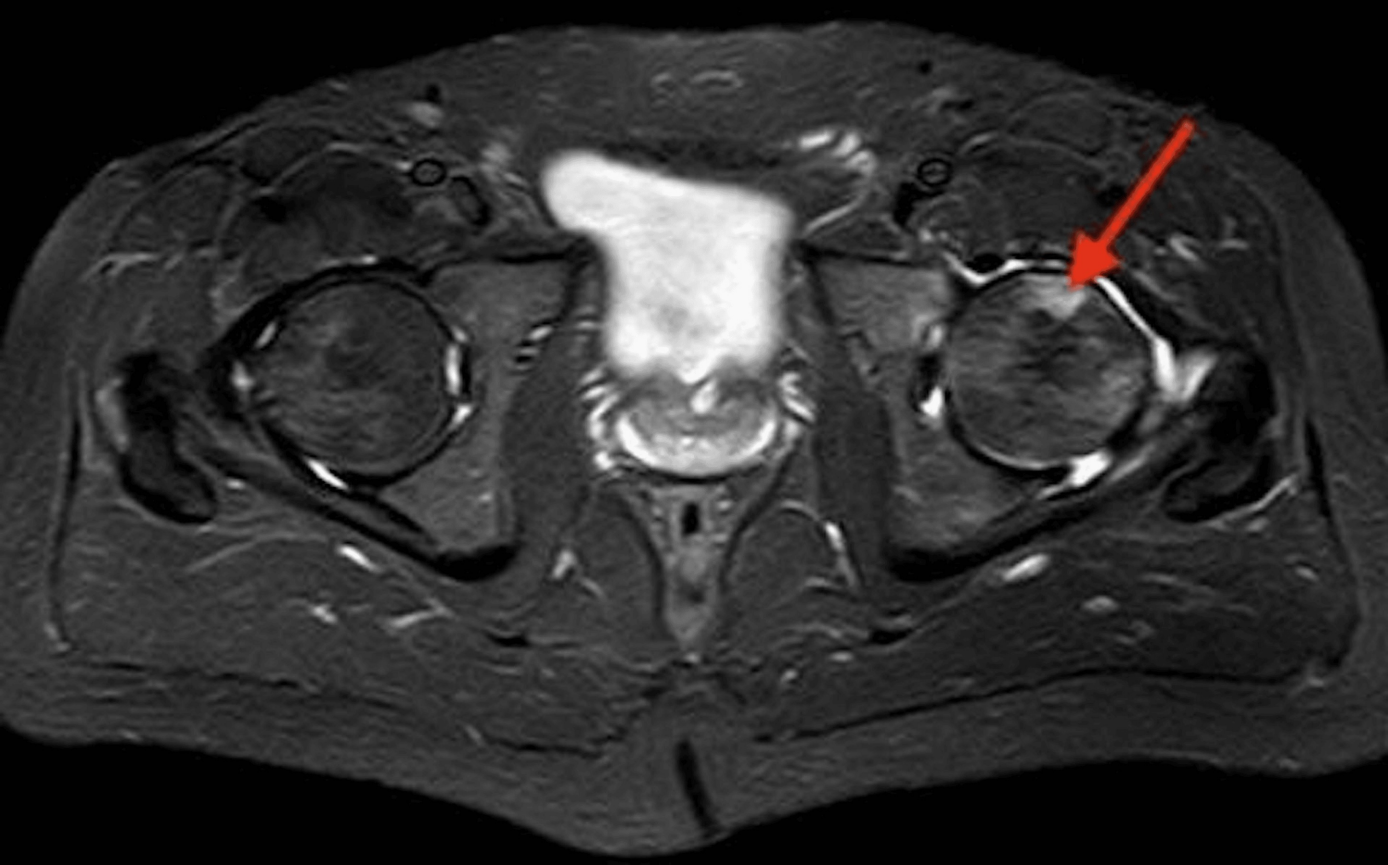
MRI of the pelvis showing normal right hip joint with bone marrow edema in the left acetabulum, femoral head, and neck Red arrow: left hip joint effusion and edema in the femoral head, neck, and acetabulum MRI: magnetic resonance imaging

**Figure 3:**
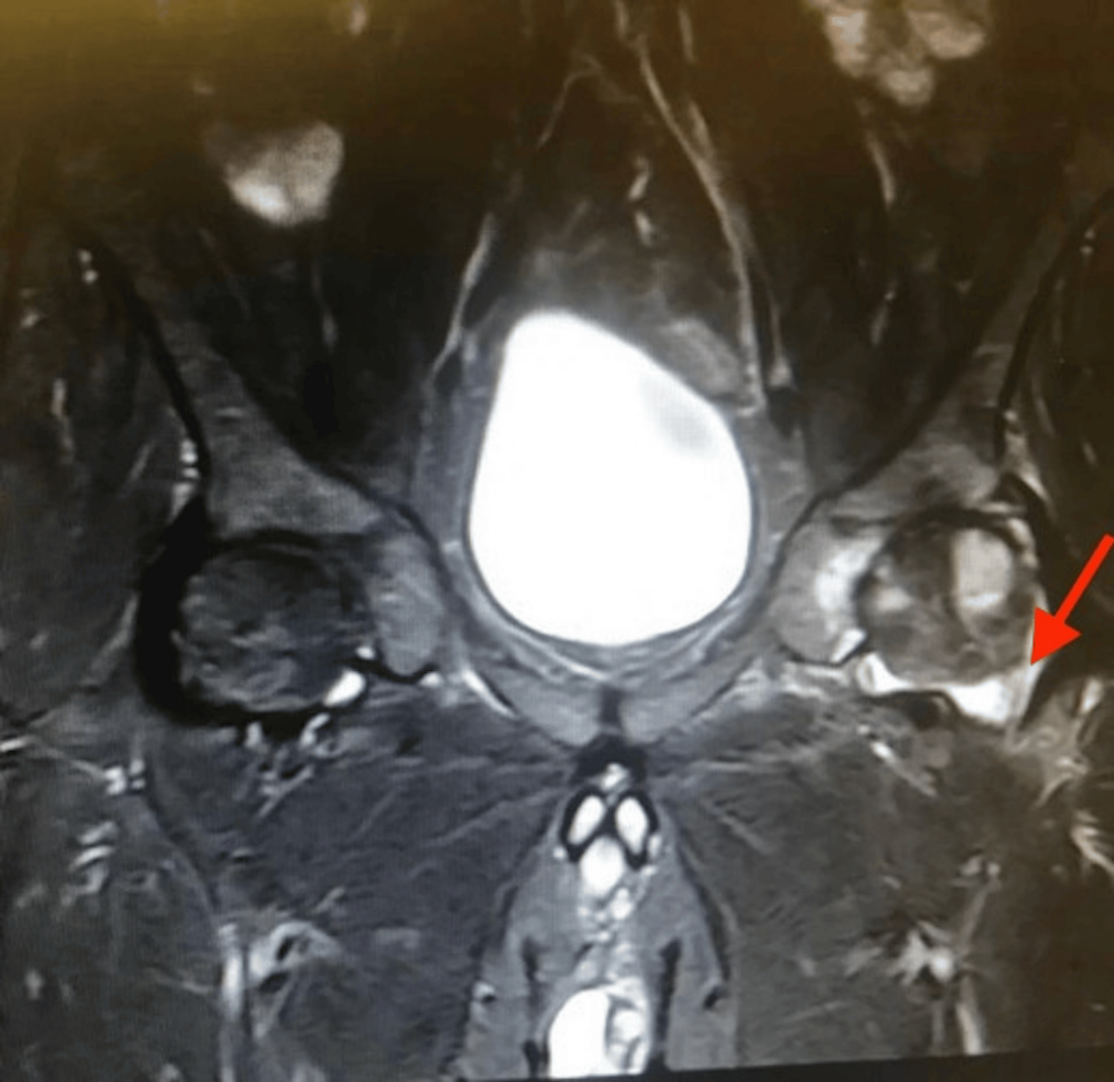
MRI showing left hip joint effusion Red arrow: effusion seen in the left hip joint MRI: magnetic resonance imaging

Further evaluation of the left hip was done by MRI to rule out left trochanteric bursitis (Figures 2-3). Only a plain MRI study without contrast was carried out as per clinical history. The alignment of bones at the left hip joint was maintained. There was no evidence of clear fracture or subluxation. There were associated patchy scattered areas of bone marrow edema (BME) of variable degree at the left femoral head more marked at the supero-lateral aspect associated with edema of the left femoral neck extending to the inter-trochanteric region.

No clear evidence of fracture or stress fractures, bony erosions, or osteochondral defects were seen. There were no clear MR signs of osteonecrosis of the femoral head. There was mild to moderate hip joint effusion. The joint space was still preserved. The articular cartilage, transverse ligament, ligamentum teres, and labrum were intact. Muscles, tendons, and related bursae around the left hip joint were preserved. There was no MR evidence of left trochanteric bursitis. Sacroiliac joints, symphysis pubis, and sacrum appeared unremarkable. Degenerative disc changes were seen in lower lumbar discs. The right hip joint along with the pelvic viscera grossly appeared normal. While the present study was sub-optimal in the evaluation of the scrotum, there was an indication of probable hydrocele on either side. Bone scintigraphy was done, which demonstrated markedly increased homogeneous uptake in the femoral head.

**Figure 4:**
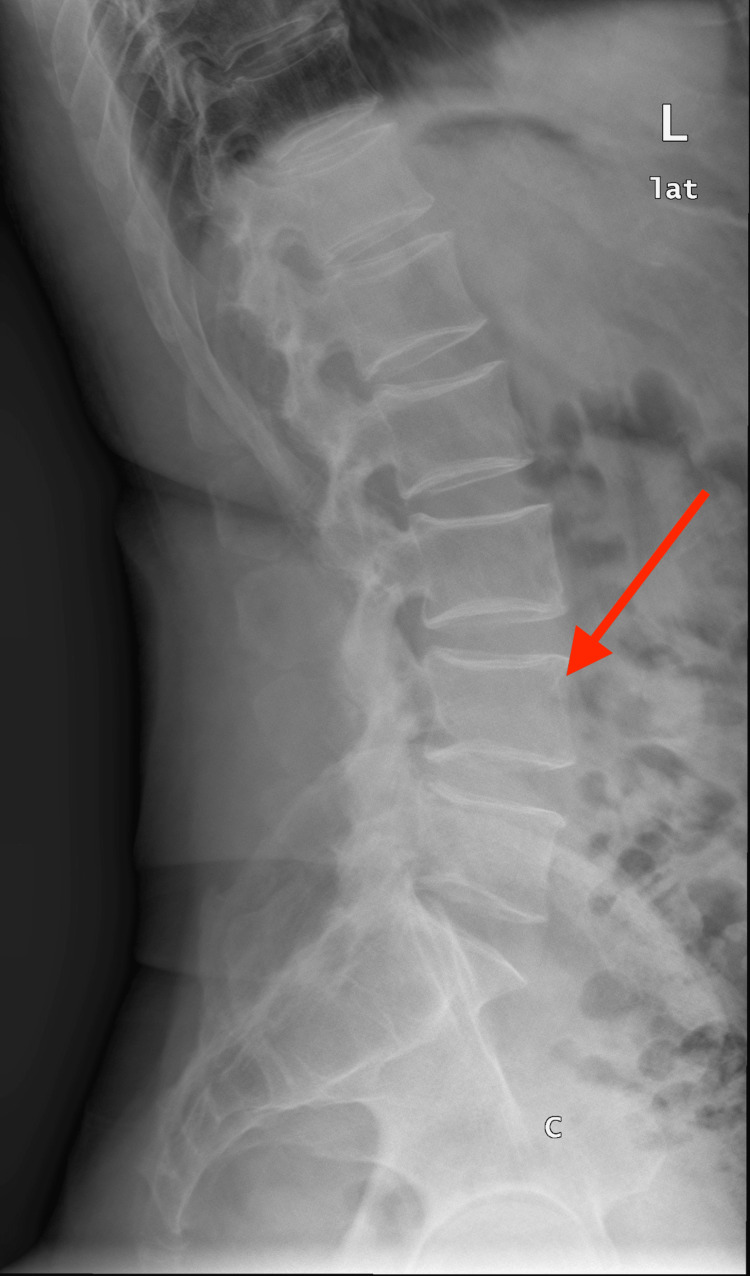
X-ray of the lumbar spine showing degenerative disc changes at the lower lumbar discs Red arrow: degenerative changes in the lower lumbar discs

Given the lower back pain as well as incidental findings seen in the MRI of the left hip of the patient, a plain study of the lumbosacral spine was conducted to rule out disc prolapse. The findings showed subtle insignificant lumbar scoliosis with rightward convexity. The lumbar vertebrae and posterior elements were intact and showed normal marrow signals. At D12/ L1, there was moderate degenerative disc change without any posterior disc bulge or disc protrusion. Figure 4 shows mild degenerative disc changes at L1/L2, L2/L3, and L3/L4 without any posterior disc bulge or disc protrusion. At L4/L5, there were moderate changes of disc desiccation with preserved disc height, left postero-lateral annular fissure, posterior diffuse disc bulge indenting thecal sac and also encroaching neural foramina and probably impinging on exiting nerve roots, particularly on the right side.

There was bilateral mild hypertrophy of ligamentum flava and facet joints. At L5/S1, there were mild degenerative disc changes, posterior right paracentral annular fissure, and posterior diffuse annular disc bulge indenting thecal sac without any focal neural impingement effects on exiting nerve roots. The conus medullaris was normal in location (at L1 lower border level) and displayed normal anatomical configuration and uniform parenchymal signal intensity. The Sl joints grossly appeared normal and the paraspinal muscles were intact. Significant BME at the left acetabulum and patchy BME at the head of the left femur with left hip joint effusion were observed. There was an incidental finding of comparatively excessive left renal sinus fat.

The patient was managed with nonsteroidal anti-inflammatory drugs (NSAIDs) and other pain relievers to help manage the pain and inflammation. He was advised to avoid activities that exacerbated the pain, such as walking or climbing stairs, until the symptoms improved. Physical therapy and exercises were also recommended to help improve flexibility, strength, and range of motion, which may alleviate pain and improve overall function. The use of crutches or a walker to reduce weight-bearing on the affected hip joint was also advised.

The patient was prescribed bisphosphonates or other osteoporosis medications such as denosumab, hormone replacement therapy, or parathyroid hormone analogs for the next three to six months with regular follow-up to improve bone density and prevent fractures. Rest, recovery, and allowing time for the hip joint to heal and recover through periods of rest were also encouraged. Regular follow-up appointments were required and the patient was advised to adjust the treatment plan if needed. However, he was lost to follow-up, and no repeat imaging was performed after the initial evaluation. This limits the ability to assess the progression of the findings; however, the initial imaging provides important diagnostic information.

## Discussion

TOH is an uncommon, self-limiting condition that presents with acute hip pain and localized bone demineralization, predominantly affecting middle-aged men and pregnant women in their third trimester, with a male-female ratio of 3:1 [1,2]. The exact cause of this condition remains unknown, and several theories have been proposed to explain it. The etiology of TOH may involve microvascular injury, nontraumatic reflex sympathetic dystrophy, metabolic changes, viral infection, and neurological and endocrine factors. Viral infections such as HIV, hepatitis C, and parvovirus B19 could lead to increased osteoclastic bone resorption, causing stress fractures in the femoral head and resulting in hip pain [3].

The presence of BME surrounding the femoral hip has led researchers to consider ischemia as a potential underlying cause in these patients, possibly due to a disturbance in venous drainage [4]. Angiographic and scintigraphic studies have supported this theory, showing dilated nutrient vessels in the femoral head, which could result in increased perfusion in the affected area, suggesting ischemia as a causative factor [5]. Some researchers believe that TOH may be caused by a transient ischemic episode leading to a contained area of cell necrosis [6]. It has been described as an early reversible phase of other similar conditions, such as avascular osteonecrosis of the hip [7].

There are similarities between TOH and nontraumatic complex regional pain syndrome (CRPS) in clinical and radiological findings. However, TOH lacks specific cutaneous changes characteristic of reflex sympathetic dystrophy, possibly due to the deep location of the hip joint [8]. Nonetheless, we believe that TOH may be considered a subset of CRPS due to its shared features, such as an unknown cause, characteristic pain (often disproportionate to physical and radiological findings), and vasomotor dysfunction of the extremity, developing without an identifiable precipitating event. It is crucial to differentiate TOH from other conditions with similar clinical presentations, such as avascular necrosis, insufficiency fracture, infective, and inflammatory arthritis. The onset of pain in TOH is sudden and more severe than osteonecrosis. Clinically, it presents as a dull aching pain in the groin region, buttocks, or anterior aspect of the thigh. It is frequently accompanied by a limp and an antalgic gait. The pain is worse in the night and on weight bearing. Functional disability is often disproportionate to the symptoms with preservation of the range of movement [9].

The radiographic features often lag behind clinical symptoms by one to two months. Initial X-rays show focal osteopenia involving the femoral head and neck region. As the disease progresses, there might be complete effacement of the subchondral cortex of the femoral head, and sometimes, a near absence of the osseous architecture, thereby creating an optical void known as the phantom appearance’ of the femoral head. A bone scan is a sensitive but nonspecific test. There is an increased uptake with radioisotope bone scanning even before radiographic changes are visible, thereby helping in the early diagnosis of the pathology. Bone scintigram is characterized by increased uptake in all three phases, indicating a focal area of hyperemia and increased capillary permeability with an increase in osteoblastic activity. Radionuclide scanning provides an efficient screening tool for the entire body, and may also reveal the asymptomatic involvement of other body parts such as the contralateral hip. An MRI is a very sensitive test to diagnose TOH [10].

TOH is a condition that usually resolves on its own within 6-12 months, and treatment focuses on managing symptoms and providing support. NSAIDs are often prescribed to alleviate pain, along with protected weight bearing and a gradual physiotherapy regimen to improve joint function during the acute phase [11,12]. Intermittent traction can help prevent deformity associated with joint effusion. Various therapeutic agents have been discussed as potential treatments for TOH. Bisphosphonates, available in oral or intravenous forms, are considered effective in speeding up recovery. Additionally, calcitonin, administered as a nasal spray with a daily dosage of 200 IU [13], may help reduce bone loss during the acute phase of the disease [14]. Recently, iloprost, a prostacyclin analog, has shown promising results in managing painful BME of the knee joint [15]. Its effectiveness in TOH is still being evaluated, as it possesses properties that dilate blood vessels and reduce permeability in small vessels (capillaries).

To sum up, TOH is a rare condition characterized by sudden-onset hip pain and localized bone demineralization. Despite its concerning presentation and radiological findings, TOH is known to resolve on its own within a few months. The exact cause of TOH remains uncertain, with various theories proposed, including microvascular injury, reflex sympathetic dystrophy, viral infection, ischemia, and nontraumatic factors.

## Conclusions

We discussed the clinical history and radiological findings of a 58-year-old male with TOH. The patient experienced severe lower back pain radiating to the left hip, worsened by weight-bearing activities. Lab tests showed normal values for relevant biomarkers, and X-rays did not reveal any abnormalities. However, the MRI displayed BME and joint effusion in the left hip, confirming the diagnosis of TOH. The patient was not available for follow-up, and no repeat imaging studies were conducted after the initial evaluation. Future follow-up with repeat imaging would have been ideal to monitor changes, particularly in the areas of BME and joint effusion, as these findings could evolve over time. The management of TOH primarily involves conservative approaches, including pain control, rest, and physical therapy to improve joint function and reduce discomfort. In more complex cases, medications like bisphosphonates may be considered. Surgical options, such as core decompression, remain a topic of debate and are reserved for specific cases. Further research is essential to fully comprehend the underlying mechanisms of TOH and explore potential targeted treatments. Early diagnosis and appropriate management are crucial to alleviate symptoms and facilitate recovery, and clinicians should be aware of this condition to provide timely and effective care for affected patients.
